# Methylene Blue to Treat Protamine-induced Anaphylaxis Reactions. An Experimental Study in Pigs

**DOI:** 10.5935/1678-9741.20160054

**Published:** 2016

**Authors:** Agnes Afrodite S. Albuquerque, Edson A. Margarido, Antonio Carlos Menardi, Adilson Scorzoni Filho, Andrea Carla Celotto, Alfredo J. Rodrigues, Walter Vilella A. Vicente, Paulo Roberto B. Evora

**Affiliations:** 1 Laboratory of Endothelium and Cardiovascular Function; Department of Surgery and Anatomy, Faculdade de Medicina de Ribeirão Preto da Universidade de São Paulo (FMRP-USP), SP, Brazil.

**Keywords:** Protamines, Nitric Oxide, Methylene Blue, Anaphylaxis

## Abstract

**OBJECTIVE:**

To examine if methylene blue (MB) can counteract or prevent protamine (P) cardiovascular effects.

**METHODS:**

The protocol included five heparinized pig groups: Group Sham -without any drug; Group MB - MB 3 mg/kg infusion; Group P - protamine; Group P/MB - MB after protamine; Group MB/P - MB before protamine. Nitric oxide levels were obtained by the nitric oxide/ozone chemiluminescence method, performed using the Nitric Oxide Analizer 280i (Sievers, Boulder, CO, USA). Malondialdehyde plasma levels were estimated using the thiobarbiturate technique.

**RESULTS:**

1) Groups Sham and MB presented unchanged parameters; 2) Group P - a) Intravenous protamine infusion caused mean arterial pressure decrease and recovery trend after 25-30 minutes, b) Cardiac output decreased and remained stable until the end of protamine injection, and c) Sustained systemic vascular resistance increased until the end of protamine injection; 3) Methylene blue infusion after protamine (Group P/MB) - a) Marked mean arterial pressure decreased after protamine, but recovery after methylene blue injection, b) Cardiac output decreased after protamine infusion, recovering after methylene blue infusion, and c) Sustained systemic vascular resistance increased after protamine infusion and methylene blue injections; 4) Methylene blue infusion before protamine (Group MB/P) - a) Mean arterial pressure decrease was less severe with rapid recovery, b) After methylene blue, there was a progressive cardiac output increase up to protamine injection, when cardiac output decreased, and c) Sustained systemic vascular resistance decreased after protamine, followed by immediate Sustained systemic vascular resistance increase; 5) Plasma nitrite/nitrate and malondialdehyde values did not differ among the experimental groups.

**CONCLUSION:**

Reviewing these experimental results and our clinical experience, we suggest methylene blue safely prevents and treats hemodynamic protamine complications, from the endothelium function point of view.

**Table:** 

**Abbreviations, acronyms & symbols**	
CO	= Cardiac output
CVP	= Central venous pressure
MAP	= Mean arterial pressure
MB	= Methylene blue
MDA	= Malondialdehyde
NO	= Nitric oxide
NOx	= Nitrite/nitrate
PAP	= Pulmonary arterial pressure
PCP	= Pulmonary capillary pressure
PVR	= Pulmonary vascular resistance
SEM	= Standard error of the mean
SVR	= Systemic vascular resistance

## INTRODUCTION

Clinical and experimental observations prove that heparinneutralizing doses of protamine increase pulmonary artery pressures and decrease systemic blood pressure. Protamine also increases myocardial oxygen consumption, cardiac output (CO), and heart rate, and decreases systemic vascular resistance. These cardiovascular effects have clinical consequences that have justified studies in this area. Protamine adverse reactions usually can be classified into three different categories, namely: systemic hypotension, anaphylactoid reactions, and catastrophic pulmonary vasoconstriction. The precise mechanism that explains protamine-mediated systemic hypotension is unknown. Four experimental protocols performed at Mayo Clinic (Rochester, MN, USA) studied the intrinsic mechanism of protamine vasodilation. The first study reported *in vitro* systemic and coronary vasodilation after protamine infusion^[1]^. The second *in vitro* study suggested that pulmonary circulation is extensively involved in the protamine-mediated effects on endothelial function^[2]^. The third study, carried out in anesthetized dogs, reported the methylene blue (MB) and nitric oxide (NO) synthase blockers neutralization of the protamine vasodilatory effects^[3]^ The fourth study proposed that protamine also causes endothelium-dependent vasodilation in heart microvessels and conductance arteries by different mechanisms, including hyperpolarization^[4]^. Reviewing those experimental results and our clinical experience, we suggest MB as a novel approach to prevent and treat hemodynamic complications caused by the use of protamine after cardiopulmonary bypass^[5]^.

In the absence of prospective clinical trials and cumulative clinical evidence, based on the literature case reports, the present study was carried out to examine if MB can counteract or prevent protamine cardiovascular effects.

## METHODS

### Experimental design

The protocol included five heparinized pig groups: Group Sham - without any drug; Group MB - MB 3 mg/kg infusion; Group P - protamine; Group P/MB - MB after protamine; Group MB/P - MB before protamine. NO plasma levels were measured in each of the experimental groups. The procedures and handling of the animals were reviewed and approved by the Institutional Animal Care review board (Reg 142/2006).

### Animal preparation and hemodynamic parameters

Female Dalland pigs (22-26 kg) were induced to anesthesia with intramuscular administration of midazolam (15 mg/kg, Dormid^®^, Cristália Produtos Químicos Ltda., SP, Brazil) and tiletamine/zolazepam (10 mg/kg, Telazol^®^, Fort Dodge, IA, USA). Maintenance was achieved by total intravenous anesthesia using sufentanil (100 µg/h, Fastfan^®^, Cristália Produtos Químicos Ltda., SP, Brazil) and propofol (10 mg/kg/h, Propovan^®^, Cristália Produtos Químicos Ltda., SP, Brazil) delivered by syringe infusion pump (Syringe Infusion Pump, Harvard Apparatus, MA, USA). Pancuronium bromide (6 mg/h, Pancuron^®^, Cristália Produtos Químicos Ltda., SP, Brazil) was used as a muscle relaxant. Tracheostomy was performed on all animals immediately after induction of anesthesia. Volemia maintenance was achieved with intravenous infusion of sodium chloride 0.9% (5 mL/kg/h). A Swan-Ganz CCOmbo CCO/SvO_2_ 744HF75 (Edwards Lifesciences, CA, USA) catheter was placed in the right jugular vein and into the lumen of the main pulmonary artery. The left carotid artery was simultaneously catheterized. Mean arterial pressure (MAP), pulmonary arterial pressure (PAP), pulmonary capillary pressure (PCP) and central venous pressure (CVP) were recorded by the MP System 100 A (BioPac System, Inc., CA, USA). Cardiac output (CO), systemic vascular resistance (SVR) and pulmonary vascular resistance (PVR) were obtained by the Vigilance System (Edwards Lifesciences LLC, CA, USA). After instrumentation, a period of 20 minutes was allowed for anesthesia stabilization. After that, hemodynamic parameters and clinical conditions were recorded for 15 minutes.

### Statistical analysis

The hemodynamic results were expressed as mean ± standard error of the mean (SEM) and analysis of variance (Twoway ANOVA) and Bonferroni post-test. The Nitrite/Nitrate (NOx) and malondialdehyde (MDA) results were analyzed using paired T-test (Prism 5.0, GraphPad Software Inc., San Diego, CA, USA). Values are considered to be statistically significant at *P* values smaller than 0.05.

## RESULTS

### Mean arterial pressure (MAP)

**Fig. 1:**
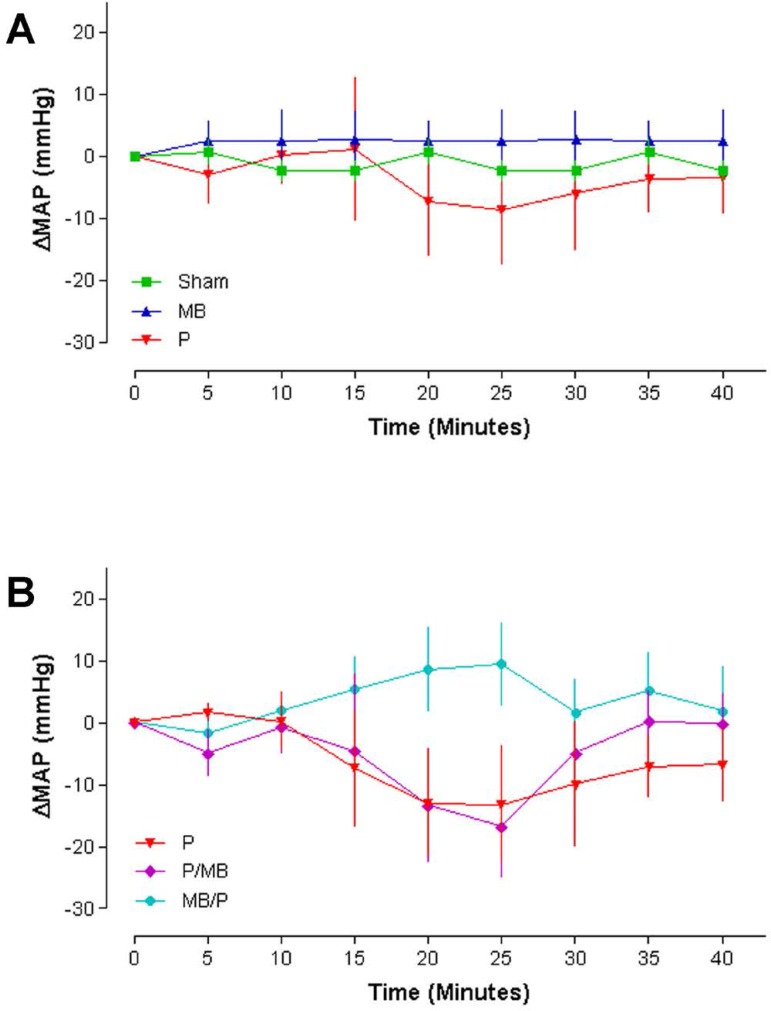
Mean arterial pressure (n=6, Two-way ANOVA. Bonferroni post-test. P<0.05). MAP=mean arterial pressure; MB=methylene blue; P=protamine

Groups Sham, MB and P showed unchanged parameters. Intravenous P infusion caused MAP drop followed by a recovery trend after 25-30 minutes. The MAP curves of Sham, P and MB were not different and the effect was considered not quite significant (*P*=0.05) (Figure 1A).

In the MB infusion before protamine group (Group MB/P), the drop in MAP was less severe, with rapid recovery. In MB infusion after protamine group (Group P/MB), marked MAP decrease after protamine was observed, with recovery after MB injection. The curves of P, P/MB and MB/P groups were not different and the effect was not significant (*P*=0.3786) (Figure 1B).

### Cardiac output (CO)

**Fig. 2:**
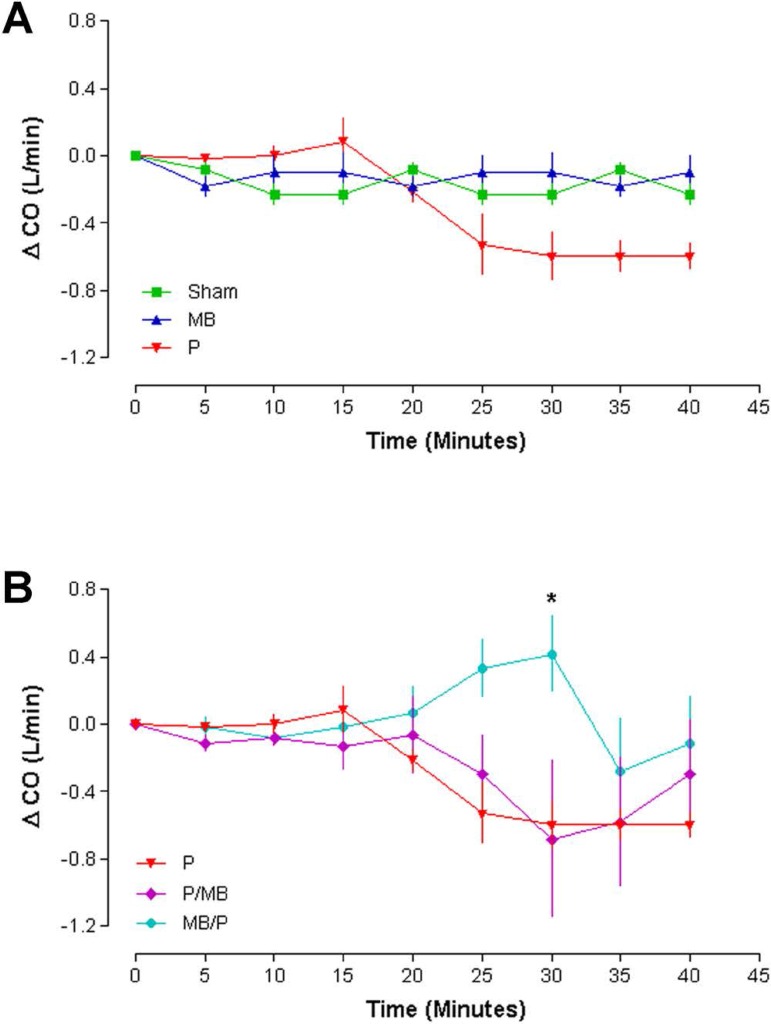
Cardiac output (n=6, Two-way ANOVA. Bonferroni post-test. P<.05). CO=cardiac output; MB=methylene blue; P=protamine

Groups Sham, P and MB showed unchanged parameters (n=6; *P*>0.05). The statistical significance was borderline and the software pointed out that a larger number of animals would improve the data. In Group P, CO decreased and remained stable until the end of the protamine injection (Figure 2A).

In Group P/MB, CO decreased after protamine infusion, recovering after MB infusion. In the Protamine after MB group, there was a progressive CO increase to the time of protamine injection, when CO decreased. In addition, the curves of *P*, P/MB and MB/P were different and presented statistical difference (*P*<0.05) between groups P and MB/P at 30 minutes (Figure 2B).

### Systemic vascular resistance (SVR)

**Fig. 3:**
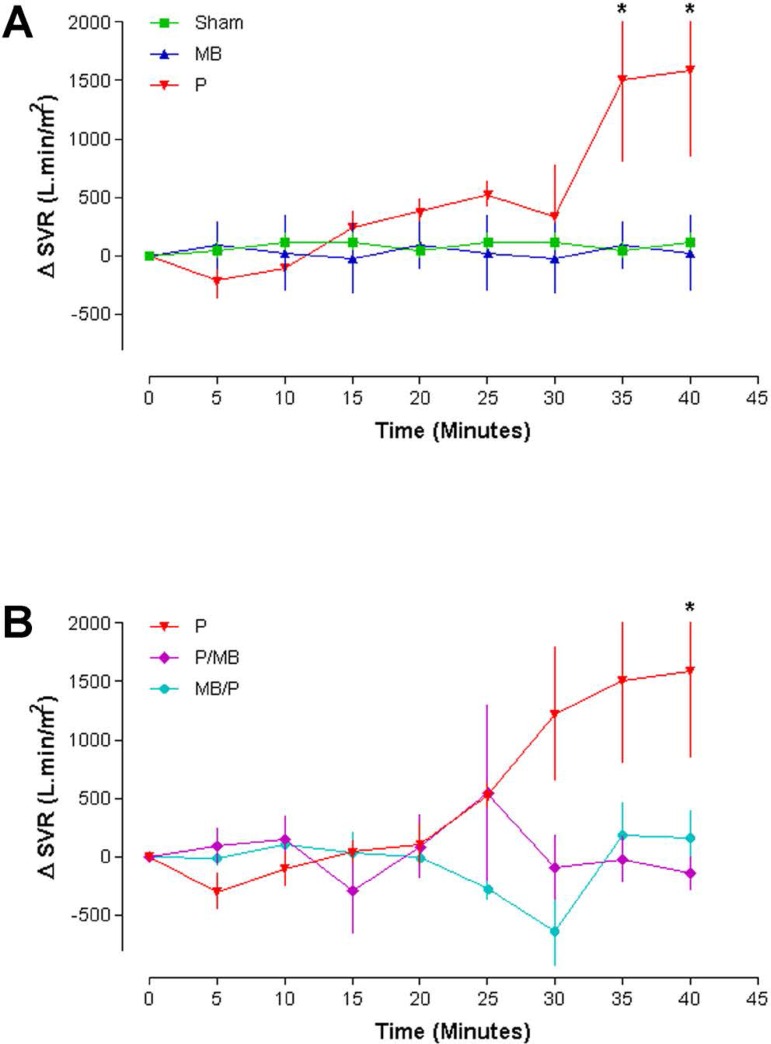
Systemic vascular resistance (n=6, Two-way ANOVA. Bonferroni post-test. P<0.05). SVR=systemic vascular resistance; MB=methylene blue; P=protamine

Groups Sham and MB showed unchanged parameters. Group P sustained an SVR increase until the end of the protamine injection. The SVR curves of Sham, P and MB were different and presented statistical difference (*P*<0.01) between groups P and Sham at 35 and 40 minutes (Figure 3A).

Group P/MB sustained SVR increase after P and MB injections and in Group MB/P, the SVR dropped after protamine followed by immediate SVR increase. Moreover, there was statistical difference (*P*<0.05) between group P *versus* P/MB and P *versus* MB/P at 40 minutes (Figure 3B).

### Pulmonary arterial pressure (PAP)

**Fig. 4:**
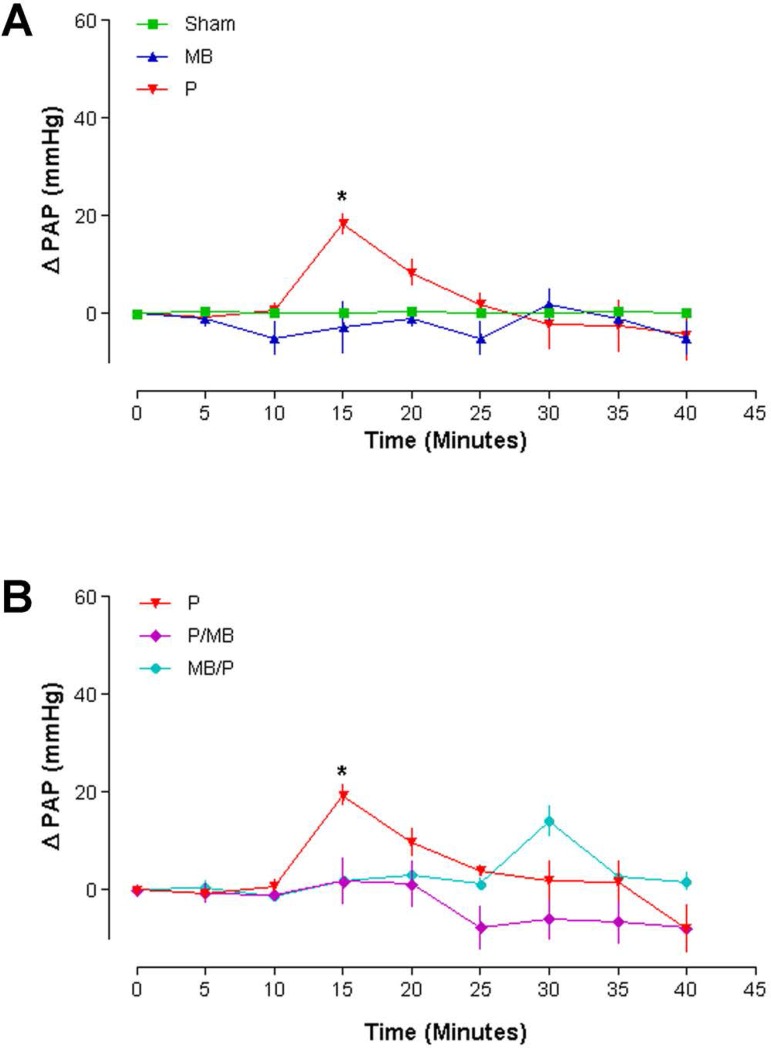
Pulmonary arterial pressure (n=6, Two-way ANOVA. Bonferroni post-test. P<0.05). PAP=pulmonary artery pressure; MB=methylene blue; P=protamine

The PAP curves in the Sham, P and MB groups showed a statistically significant increase (*P*<0.001) after protamine injection (15 minutes), followed by immediate decrease (Figure 4A). Also, the curves of Groups P, P/MB and MB/P showed statistically significant difference (*P*<0.01) between Group P versus P/MB and P *versus* MB/P at 15 minutes (Figure 4B).

### Central venous pressure (CVP)

**Fig. 5:**
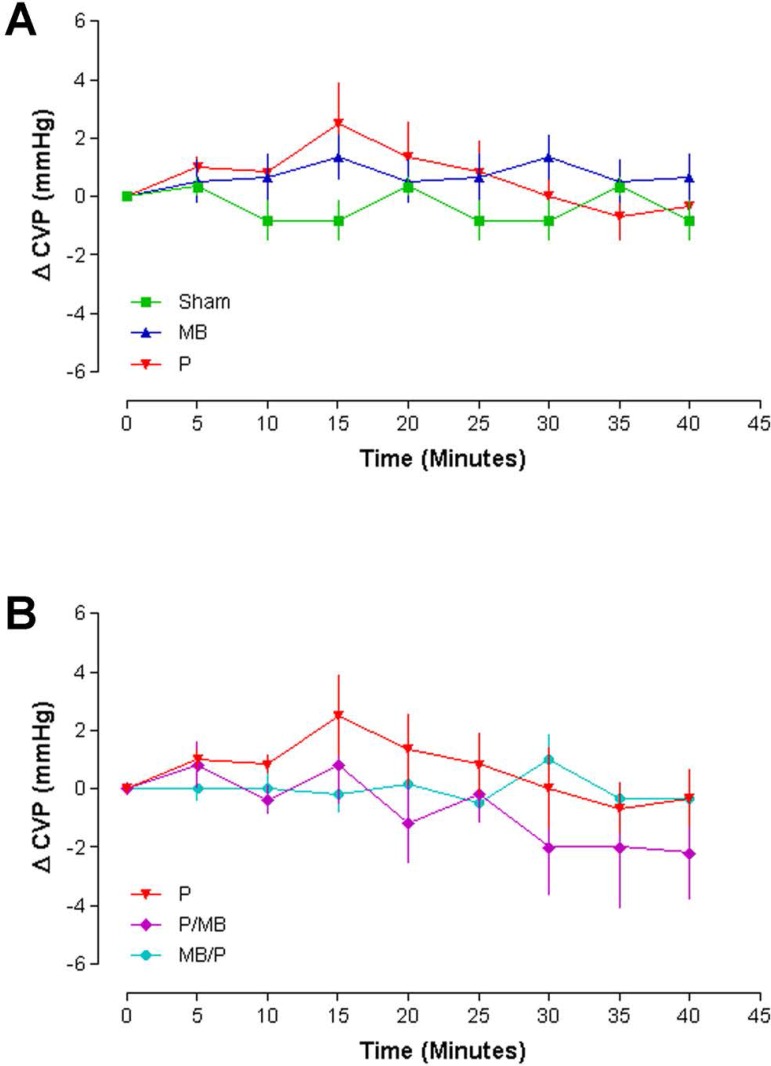
Central venous pressure (n=6, Two-way ANOVA. Bonferroni post-test. P<0.05). CVP=central venous pressure; MB=methylene blue; P=protamine

The CVP curves of Sham and MB remained stable during 40 minutes of experiment. On the other hand, Group P presented an increase at minute 15, followed by immediate decrease (Figure 5A). The curve in the MB/P group remained stable during 40 minutes of experiment and, P and P/MB presented an increase at minute 15, followed by immediate decrease (Figure 5B).

### Plasma nitrite/nitrate (NOx)

**Fig. 6:**
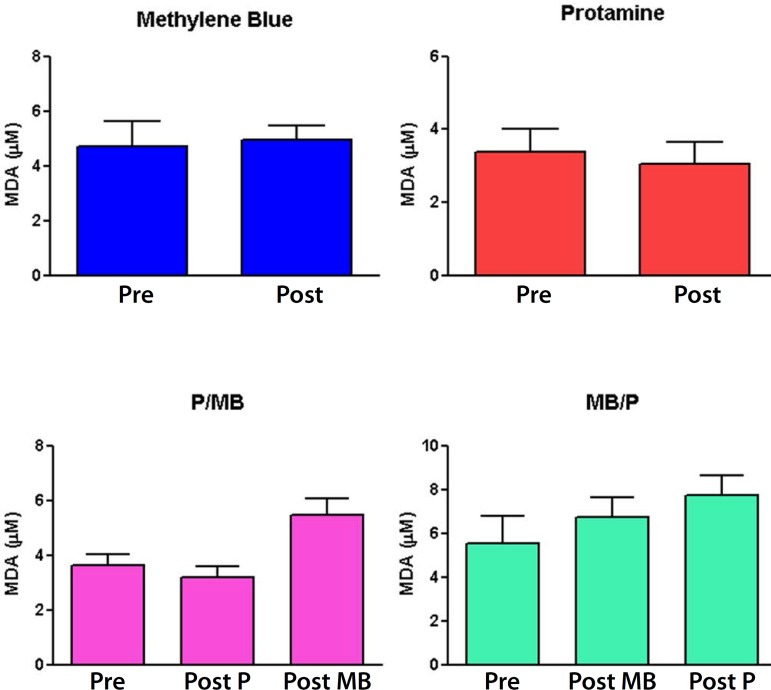
Plasma Nitrite/Nitrate (NOx) concentrations (n=6; t test. P<0.05).

Plasma NOx concentrations did not present statistically significant difference (Figure 6).

### Malondialdehyde (MDA)

**Fig. 7:**
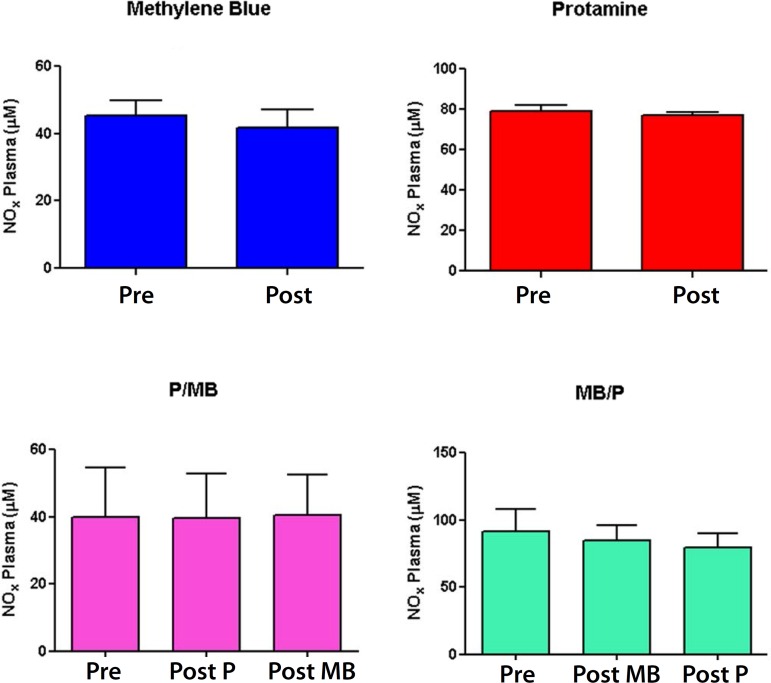
Malondialdehyde concentrations (n = 6; t test. P<0.05).

MDA concentrations did not present statistically significant difference (Figure 7).

## DISCUSSION

Heparin/protamine interaction is a topic of interest due to its use during cardiopulmonary bypass. These drugs are prescribed to more than 2,000,000 patients every year. From clinical and experimental data, heparin-neutralizing doses of protamine increase pulmonary artery pressures and decrease systemic blood pressure, myocardial oxygen consumption, CO, heart rate, and SVR. Those cardiovascular effects have clinical consequences that justified studies in this area.

Transitory hypotension in animals after protamine infusion has been observed as an experimental effect for > 50 years^[6-8]^. However, until the development of cardiac surgery, protamineinduced hypotension was an experimental finding of little clinical relevance. As protamine became largely used in clinical and surgical procedures, its reactions frequently led to severe systemic hypotension, pulmonary hypertension, and shock^[9-13]^. Those consequences can be potentially dangerous, especially immediately after cardiopulmonary bypass, when intravascular volumes are not constant and cardiac function may be impaired.

Protamine can also cause hemodynamic disturbance by means of anaphylactic reactions^[6,14,15]^. Indeed, Horrow^[15]^ classified protamine adverse reactions in three different categories: systemic hypotension, anaphylactoid reactions, and catastrophic pulmonary vasoconstriction.

The precise mechanism that explains protamine-mediated systemic hypotension is unknown. However, it is suggested that protamine decreases peripheral vascular resistance rather than depressing myocardial function^[9,11,16,17]^. Protamine sulfate (either as a free drug or complexed with heparin) binds to an unidentified endothelial cell receptor that mediates the conversion of L-arginine to NO. NO released abluminally activates soluble guanylate cyclase in the vascular smooth muscle to induce cyclic GMP (cGMP)-mediated relaxation (vasodilation). This results in a decrease in peripheral vascular resistance and hypotension. NO released luminally would promote thrombolysis and inhibit platelet adhesion in the blood vessel^[1]^. Against this hypothesis, it is important to report that Castresana et al.^[17]^, using pig vascular smooth muscle cell culture experiments, showed that protamine does not alter the responses of the intracellular second messengers, cGMP and cAMP, to the vasodilators sodium nitroprusside, atrial natriuretic peptide, isoproterenol, and forskolin. Those results do not support the hypothesis that protamine sensitizes vascular smooth muscle cells to the NO/endothelium-derived relaxing factor. Favorable to the NO/endothelium-dependent mechanism, MB has been successfully used to reverse protamine vasoplegic reactions in humans^[18-20]^.

For the endothelium-dependent vasodilatation in the pulmonary artery, a proposed mechanism suggests that protamine sulfate binds to an unidentified endothelial cell receptor to induce NO production from the amino acid L-arginine. NO then diffuses to the underlying vascular smooth muscle to induce relaxation (vasodilatation). NO released into the lumen would promote thombrolysis and inhibit platelet adhesion in the blood vessel. The conversion of L-arginine to NO can be inhibited by NG-monomethyl-L-arginine (L-NMMA), a methylated form of L-arginine. In addition, differently from the systemic circulation, heparin can inhibit the ability of protamine to induce NO, presumably by preventing the binding of protamine to receptor^[2]^. Favorable to this hypothesis is the use of inhaled NO to control pulmonary hypertension^[21-23]^.

Plasma NOx and indirect free radical activity estimated by measuring MDA levels surprisingly did not show differences. Probably, the experimental time (40 minutes) was not enough for the laboratory techniques to detect the possible stimulus for NO release and free radical (lipidperoxidation) activity.

In conclusion, based on the results described, the following can be stated: 1) Individual MB infusion did not change MAP, CO and SVR values whereas MB injected before protamine attenuated the hypotension with rapid recovery and when injected after protamine, it reversed the marked hypotension; 2) CO decreased after protamine infusion, recovering after post-MB infusion, and MB infusion before protamine caused a progressive CO increase, followed by attenuation at the time of protamine injection; and 3) There was sustained SVR increase until the end of protamine injection, decreased SVR after protamine, followed by immediate SVR increase when MB was injected before protamine, and sustained SVR increase after protamine and post MB injections. Reviewing these experimental results and our clinical experience, we suggest that MB prevents and treats hemodynamic protamine complications.

Study limitations. It is mandatory to emphasize that the animals were not under cardiopulmonary bypass that causes systemic inflammatory reaction, since this reaction should exacerbate the hemodynamic effects of protamine. Other relevant limitation is the number of animals used for each group (n=6), except for borderline statistical significances, the statistical software pointed out that a larger number of animals would improve the data.

**Table:** 

**Authors' roles & responsibilities**	
AASA	Conception and study design; analysis and/or data interpretation; statistical analysis; manuscript writing or critical review of its content; final manuscript approval
EAM	Conception and study design; analysis and/or data interpretation; final manuscript approval
ACM	Conception and study design; analysis and/or data interpretation; final manuscript approval
ASF	Conception and study design; analysis and/or data interpretation; final manuscript approval
ACC	Conception and study design; analysis and/or data interpretation; final manuscript approval
AJR	Conception and study design; analysis and/or data interpretation; final manuscript approval
WVAV	Conception and study design; analysis and/or data interpretation; final manuscript approval
PRBE	Conception and study design; analysis and/or data interpretation; statistical analysis; manuscript writing or critical review of its content; final manuscript approval
